# Geographically weighted regression model for physical, social, and economic factors affecting the COVID-19 pandemic spreading

**DOI:** 10.1007/s11356-022-18564-w

**Published:** 2022-03-04

**Authors:** Ihsan Abbas Jasim, Moheb Kamil Fileeh, Mustafa A. Ebrahhem, Laheab A. Al-Maliki, Sohaib K. Al-Mamoori, Nadhir Al-Ansari

**Affiliations:** 1 Department of Architecture Engineering, Wasit University, Al Kut, Iraq; 2 Center of Urban and Regional Planning for Postgraduate Studies, Department of Urban Planning, University of Baghdad, Baghdad, Iraq; 3 Department of Regional Planning, Faculty of Physical Planning, University of Kufa, Najaf, Iraq; 4 Department of Environmental Planning, Faculty of Physical Planning, University of Kufa, Najaf, Iraq; 5 Department of Civil, Environmental and Natural Resources Engineering, Lulea University of Technology, Lulea, Sweden

**Keywords:** COVID-19, Geographically weighted regression, Pandemic, Spatial relations, Level of urbanization, Level of movement and accessibility

## Abstract

This study aims to analyze the spatial distribution of the epidemic spread and the role of the physical, social, and economic characteristics in this spreading. A geographically weighted regression (GWR) model was built within a GIS environment using infection data monitored by the Iraqi Ministry of Health records for 10 months from March to December 2020. The factors adopted in this model are the size of urban interaction areas and human gatherings, movement level and accessibility, and the volume of public services and facilities that attract people. The results show that it would be possible to deal with each administrative unit in proportion to its circumstances in light of the factors that appear in it. So, there will not be a single treatment for all areas with different urban characteristics, which sometimes helps not to stop social and economic life due to the imposition of a comprehensive ban on movement and activities. Therefore, there will be other supportive policies other than the ban, depending on the urban indicators for each region, such as reducing external movement from it or relying on preventing public activities only.

## Introduction

The COVID-19 pandemic has become a worldwide health problem for its high transmission rate and quick expansion worldwide, and the United Nations has classified the epidemic as a human, societal, and economic crisis. Although the pandemic posed a challenge to all local and global societies and suffered many important considerations (Luo et al. 2021; Mohsen et al. 2021), the socio-economic consequences are particularly evident in developing countries (Bilal et al. 2020; Bilal et al. 2021a, b; Lak et al. 2021). Researchers all over the globe have been interested in understanding the pandemic’s driving mechanisms and spatiotemporal transmission patterns (Ismael et al. 2021; Sarwar et al. 2021). These researchers intended to establish methods for pandemic prevention and control by identifying risk hotspots and emphasizing risk variables that may contribute to its spread in cities, among other things (Bilal et al. 2021b; Rasheed et al. 2021). Furthermore, the variables related to the spreading rate were analyzed using statistical models (El Aferni et al. 2020; Liu et al. 2021a, b) to investigate the relationship between the climatic factors (Ahmadi et al. 2020; Bashir et al. 2020a, b, c ; Bilal et al. 2021a; Fareed et al. 2020; Iqbal et al. 2020; Méndez-Arriaga, 2020; Shahzad et al. 2020a, b), the environmental factors (Bashir et al. 2020c; Han et al. 2021; Kareem et al. 2021; Liu et al. 2021a, b; Mollalo et al. 2020), and the biological and epidemiological factors (Espejo et al. 2020; Hashim et al. 2021) with the COVID-19 pandemic.

Although there are numerous studies to determine the impact of various socio-economic (Stojkoski et al. 2020a), environmental, and climatic factors (Amin and Amin 2020) that helped us better comprehend the pandemic’s distribution patterns and dynamics. However, there are still certain holes and deficiencies that must be addressed (Bashir et al. 2020b; Kareem and Al-Azzawi 2021; Kim and Bostwick 2020). Despite the health awareness and medical guidelines followed, the continuing spread of the COVID-19 pandemic made us headed to the search for links between the causes of the spread of the pandemic and society’s physical, social, and economic characteristics. GWR was utilized to explore the influence of different factors on the spatial distribution of COVID-19 cases, assuming that identifying any of these relationships may help in the future to limit the spread of the pandemic despite the applications of total and partial bans on movement. For all that, the policymakers must bring about significant changes in environmental regulations to protect current environmental changes.

Therefore, the main objective of this study is to investigate and identify the relationship between the infection number and the urban elements in each district. This study will help search for other factors that may reveal the discrepancy between the distribution of epidemic infections within societies that may be highly homogeneous but differ in their urban environments. Accordingly, it is possible to come up with other solutions that differ from the current solutions that have caused significant economic and social damage.

## Materials and methods

### The selected factors

The most important thing that previous studies have proved is that the spread of the COVID-19 pandemic is due to the social closeness and direct interaction between individuals. Hence, the research tried to put criteria to study a set of factors that could affect the spread of the pandemic, namely as follows (Akin and Gözel 2020):

1. The area of the administrative unit, which makes it a variety of activities and many interconnected relationships with its neighbors from other administrative units (Kapitsinis 2020).
2. Population movement and the density of their presence, and this can be expressed through the size of the population for each part of the secondary administrative units (Jasim et al. 2021a, b; Lakshmi Priyadarsini and Suresh 2020).
3. The high population density is one of the most important factors that increase the spread of the epidemic, so urban areas with high densities will be areas that help the spread of the epidemic (Bashir et al. 2020a).
4. The level of urbanization in each administrative unit and can be adopted as an indicator of the intensity of interaction between residents of the cities of each administrative unit (Jasim et al. 2021a; Stojkoski et al. 2020b).
5. Centralization of the provision of specialized and administrative services, which is the characteristic that makes each administrative unit with a certain level of demographic attraction for individuals to fulfil their needs (Buheji and Buhaid 2020).
6. The size of the road network, the central level, and the density of the road network for each administrative unit, which makes it a terrifying passage for movement, whether to reach other areas or to receive services in this city (Lakshmi Priyadarsini and Suresh 2020).
7. The least affected areas are the areas that are within a healthy, clean, and unpolluted environment, and the affected are less, especially the areas surrounded by the agricultural or rural cover (Bilal et al. 2020).

The analysis among regions would also help better understand COVID-19 as a global model (Bilal et al. 2021a). Policymakers should focus more on environmental pollution and strengthen epidemic response strategies to reduce environmental pollution epidemic threat.

### GWR ability to explain spatial relations

GWR has been widely used to assess spatial heterogeneity in geographical data and geo-weighted regression (Brown et al. 2012). Geographically weighted regression (GWR) helps to check all spatial assumption when we analyze spatial phenomena with multi-dimensional variables effective with spatial dimension (Iyanda and Osayomi 2020).

GWR attempts to elucidate spatial differences by testing the regression model’s logical factors with variability across space (Tu and Xia 2008). The local estimate of the model parameters is obtained by weighting all adjacent observations using a distance decay function, assuming that the close measurements significantly affect the regression point than the remote measurements. So, the basic determinant factors (*R*^2^) computed for the GWR model (Brunsdon et al. 1996) are a set of local regression results including local parameter estimates (Lewandowska-Gwarda 2018). The local *R*^2^ values and the local residuals for each regression point (sampling site) are also generated. Therefore, GWR might serve as a useful tool to explore the spatially varying relationships between land use and water quality, relaxing the assumption of spatial stationarity. Fundamentally, the GWR technique captures spatial variability by calibrating a multiple regression model that allows different relationships over geographic space (Al-Mamoori and Al-Maliki 2016; Wang and Chen 2020).

### Case study

**Fig.1:**
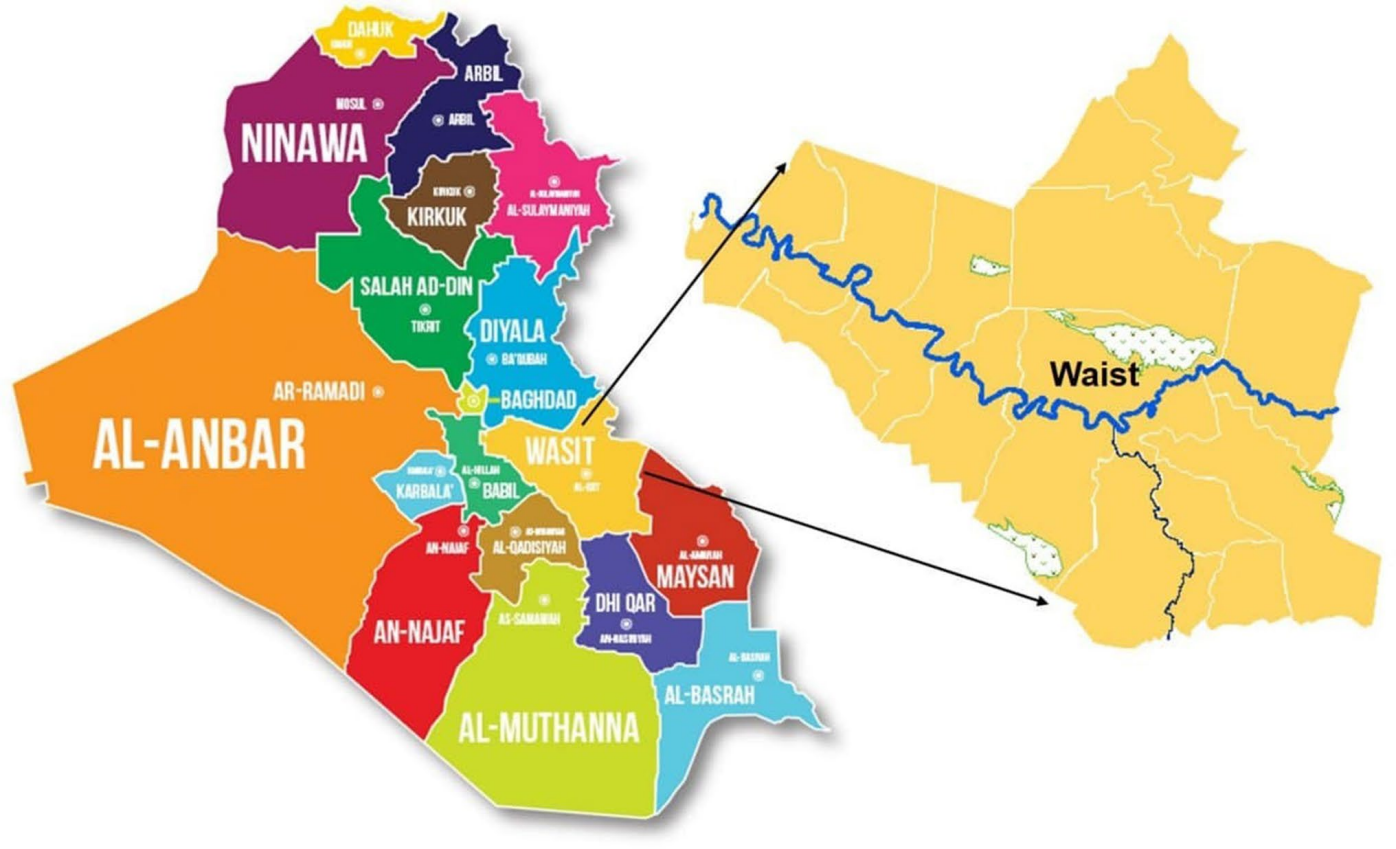
The study area location map

**Fig. 2:**
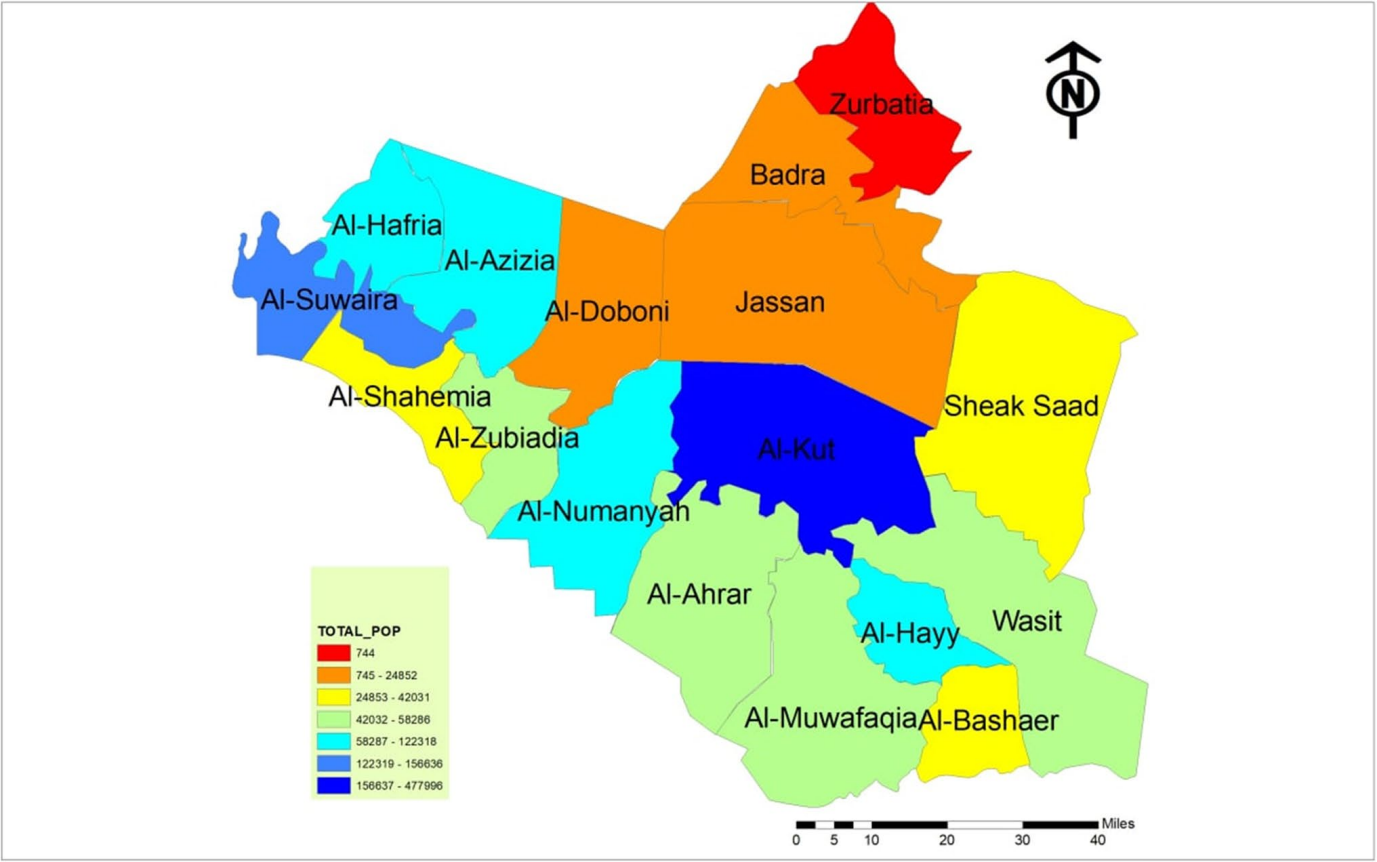
Administrative units in Wasit Governorate, Iraq

Wasit Governorate in southern Iraq was chosen to assess the relationship between socio-demographic factors and coronavirus (COVID-19) pandemic. Wasit Governorate consists of (17) administrative units with a specific hierarchical order shown in Fig. 1. It has been assumed that the GWR model can explain the relationship between the numbers of people infected with the disease as a dependent variable and the characteristics of each secondary district community for Wasit governorate. Figure 2 shows the administrative units for Wasit governorate.

**Fig. 3:**
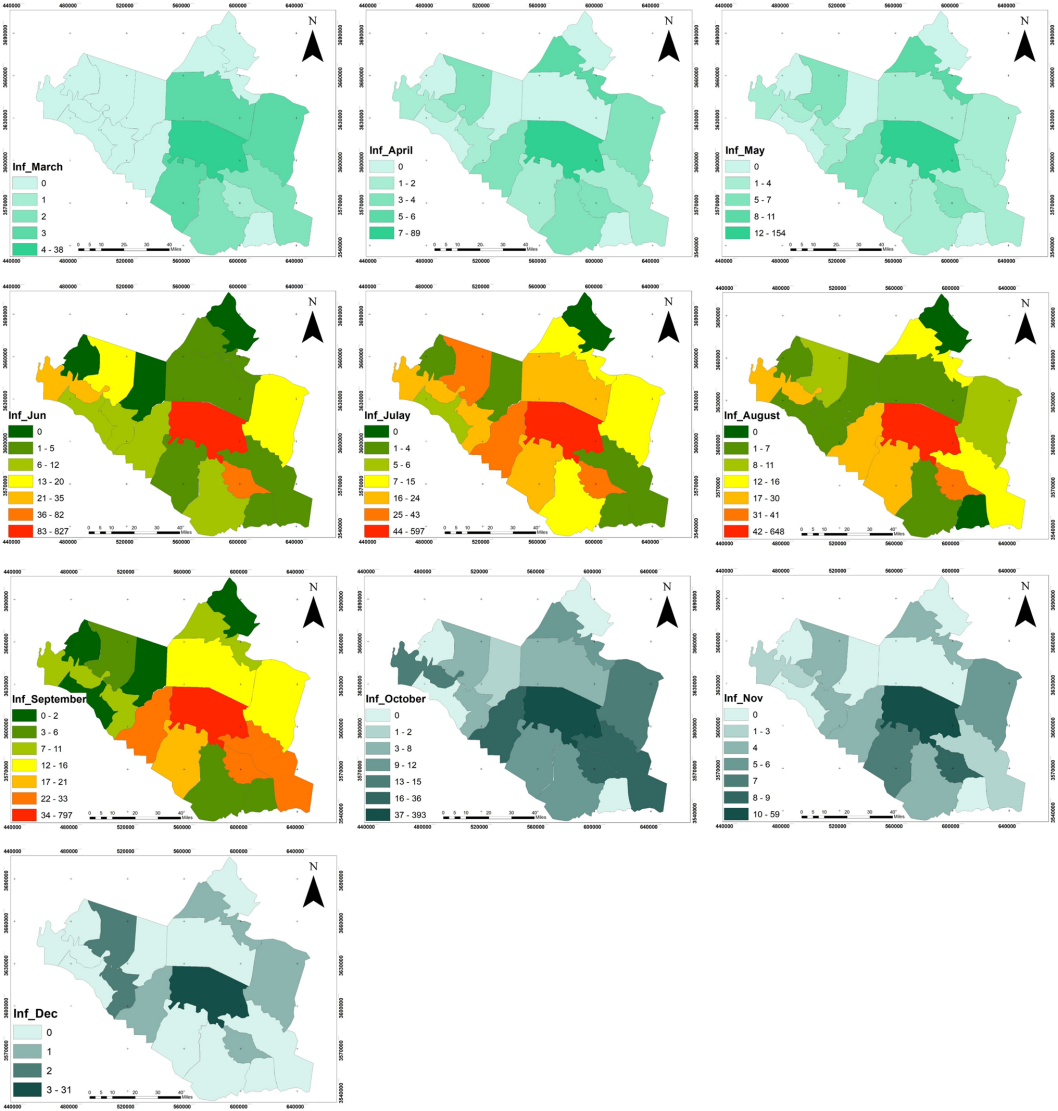
Spatial distribution of coronavirus disease in Wasit Governorate for 10 months

Figure 3 below shows the spatial distribution of coronavirus disease in Wasit Governorate, which indicates persistent infections during all months in the center of the governorate. Moreover, a fluctuation in the rate between the second and third rank for the administrative units surrounding the center of Wasit Governorate, the lowest number of COVID19 infections, was in the northern and eastern districts, especially those located on the edges of the governorate. Therefore, this spatial variation in the pandemic spread has links with a set of factors that the research will seek to test and determine their impact.

Table 1 shows the numeric values of coronavirus infections collected from Zahra General Hospital, the only hospital that served as an isolation and care center for severe cases requiring medical attention.

**Table 1:** Numeric values coronavirus infections and population in each district

District	March	April	May	June	July	Aug	Sep	Oct	Nov	Dec	TOTAL_POP
Al-Hayy	1	3	6	82	34	41	33	36	9	1	104,397
Al-Bashaer	0	0	0	2	2	0	3	0	0	0	38,499
Al-Muwafaqia	2	3	4	12	15	4	6	10	4	0	55,013
Al-Ahrar	3	2	3	3	22	30	21	12	7	0	58,286
Al-Numanyah	0	4	7	11	43	26	31	33	4	1	122,318
Wasit	2	2	4	5	4	15	32	29	3	0	50,403
Sheak Saad	3	3	4	20	15	11	16	15	6	1	42,031
Al-Kut	38	89	154	827	597	648	797	393	59	31	477,996
Jassan	3	0	2	2	22	4	14	6	0	0	12,957
Badra	0	6	11	2	13	16	10	12	4	1	16,752
Zurbatia	0	0	0	0	0	0	0	0	0	0	744
Al-Doboni	0	0	0	0	3	4	0	2	0	0	24,852
Al-Hafria	0	0	0	0	3	3	2	0	0	0	84,748
Al-Zubiadia	0	1	3	11	24	7	9	8	4	2	58,286
Al-Shahemia	0	0	0	8	6	4	0	0	0	0	38,409
Al-Suwaira	0	2	3	35	22	29	11	14	3	0	156,636
Al-Azizia	0	3	7	16	32	11	5	7	4	2	109,576

### Modelling explanatory variables

This study classified the explanatory variables into six groups: the dependent factor and the other independent factors.

**Fig. 4:**
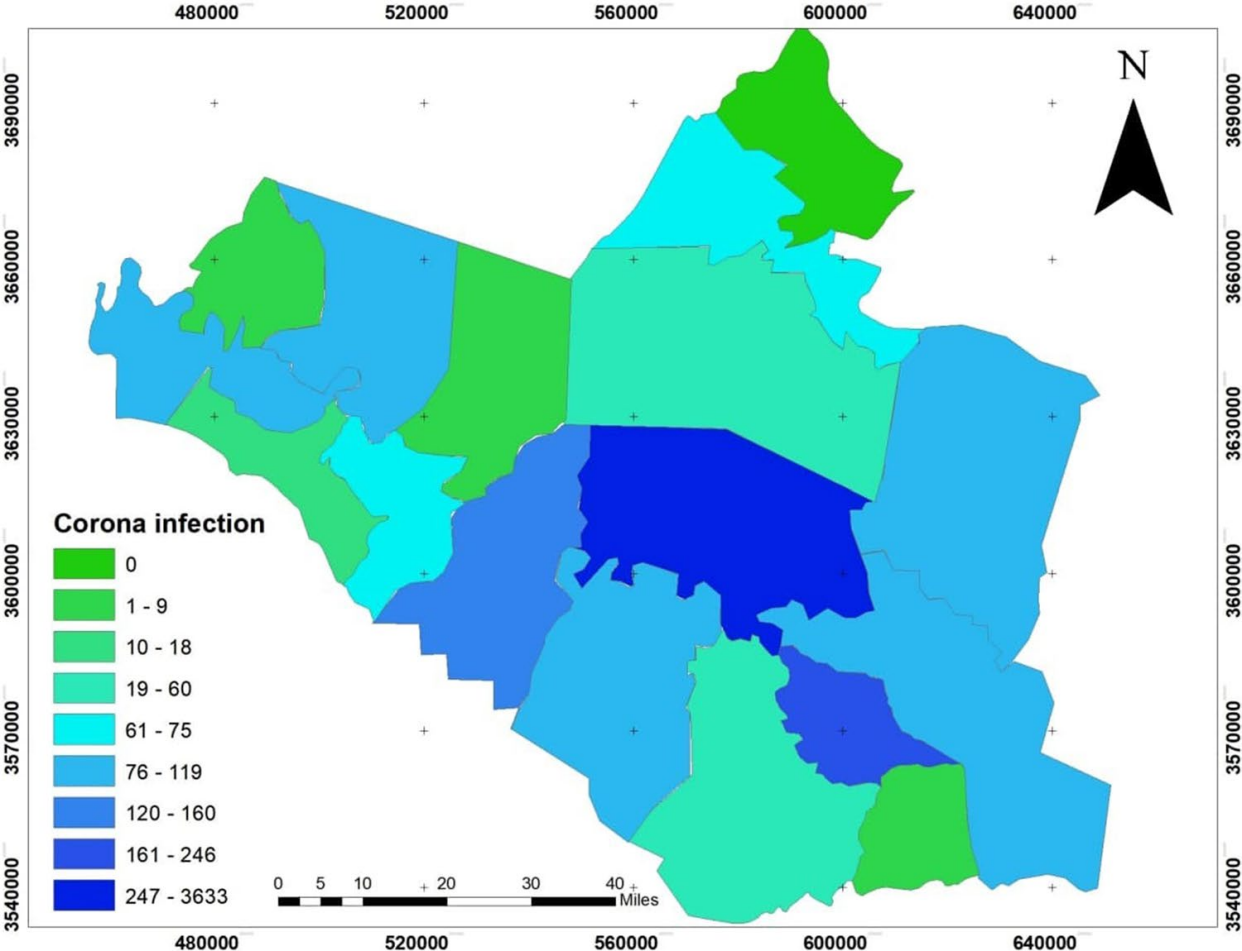
The total COVID-19 during March 2020 to December 2020

**Fig. 5:**
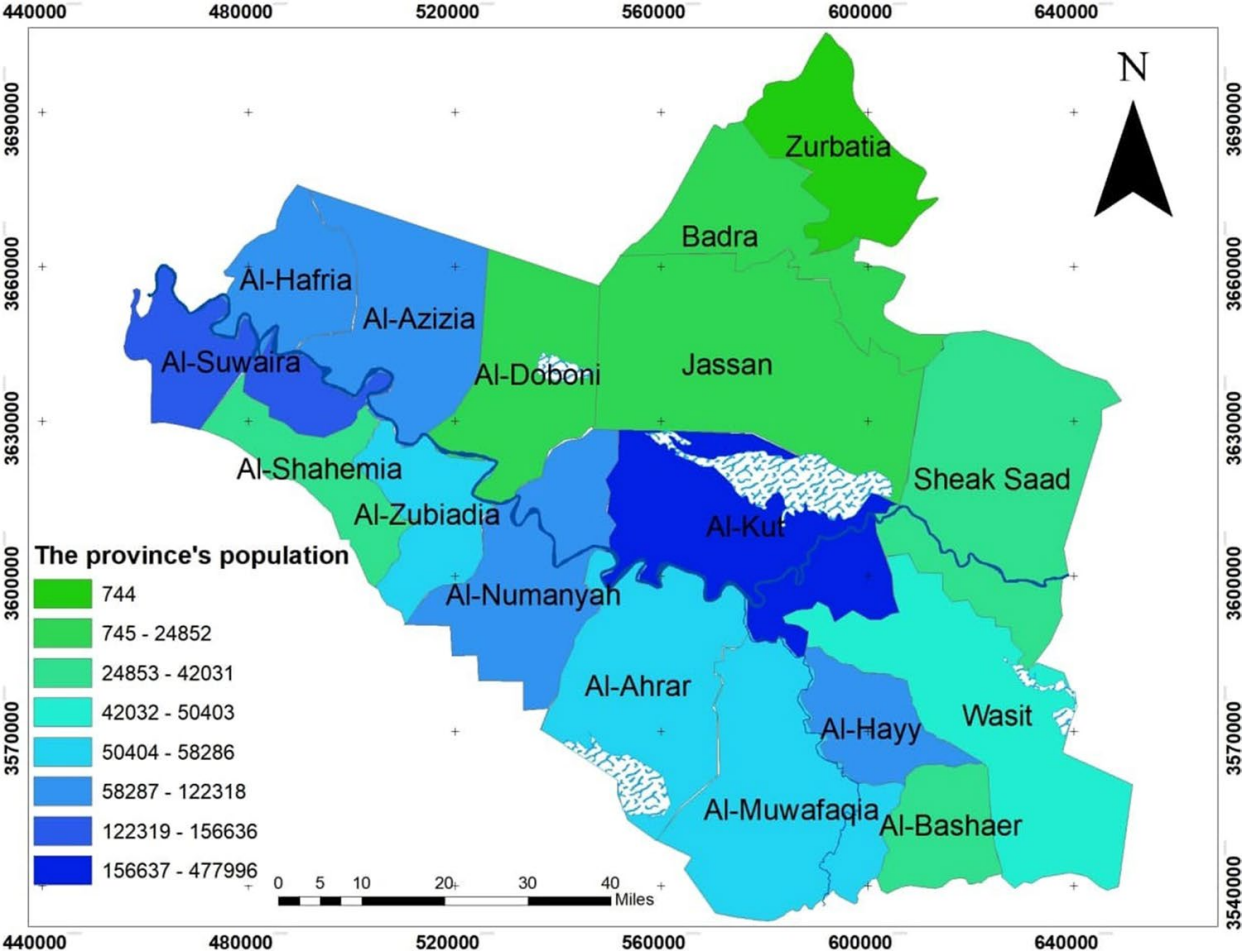
Population distribution in the Wasit Governorate

**Fig. 6:**
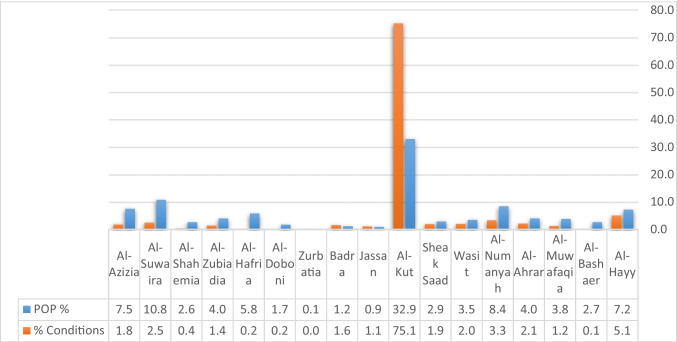
The population percentage in each district of the study area with the infection’s percentage

**Fig. 7:**
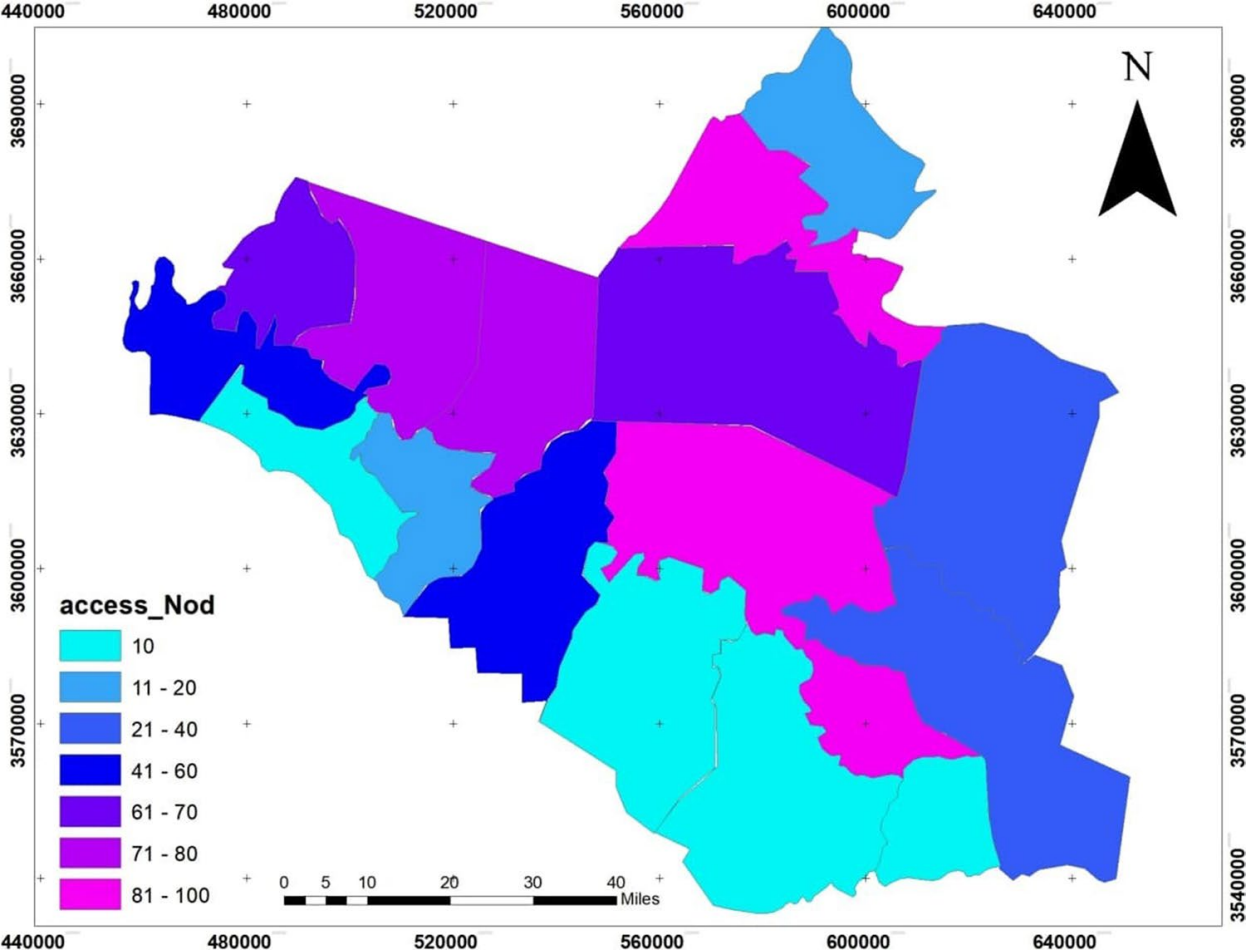
The size of the roads network in Wasit Governorate indicated by access node

**Fig. 8:**
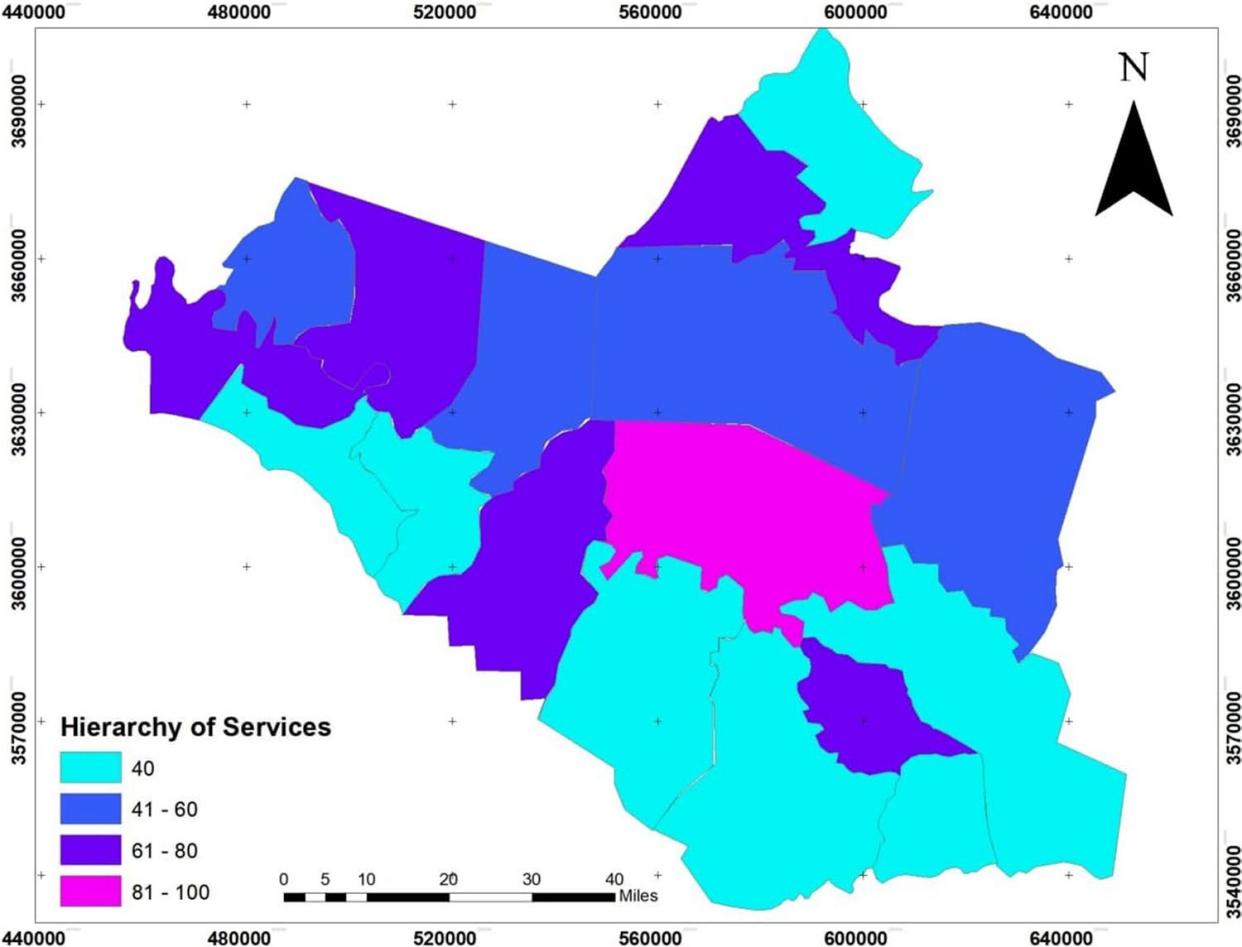
Hierarchy of services level in Wasit Governorate

**Fig. 9:**
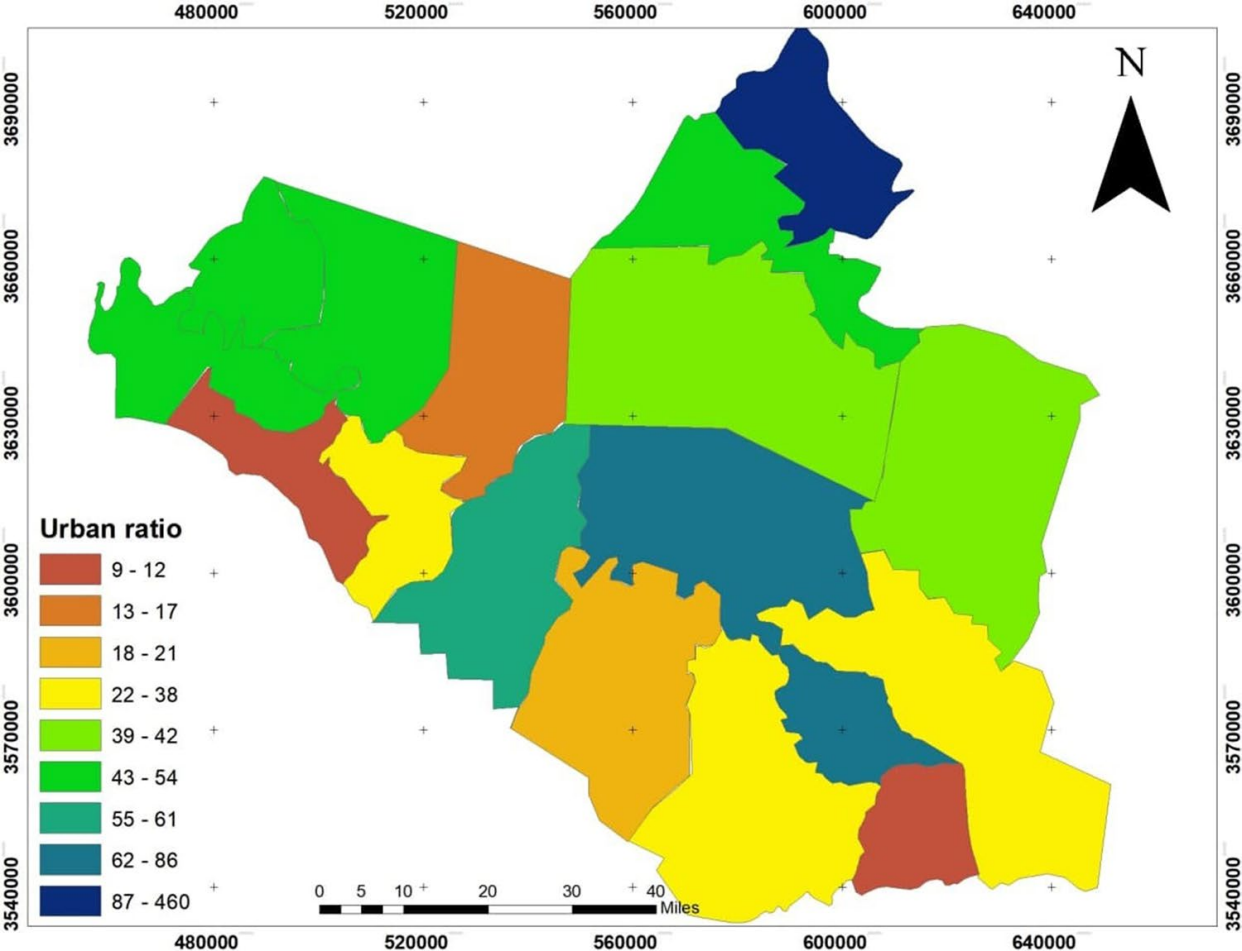
The urban ration in the district of Wasit Governorate

The logical relationship of the GWR model was formulated, which consisted of the number of people with coronavirus disease as a dependent variable and between the variables of the five factors; these variables are occurring as explanatory for the dependent one, so research collecting data for variables for GWR model are as follows:

1. The dependent variables: the infection size of COVID-19 during 10 months started from March 2020 to December 2020, see Fig. 4, which builds on the data from the Iraqi Ministry of Health. The infection is centered in the middle of Wasit Governorate, at Kut district, and a little lighter on the governorate outskirts.
2. The area of all administrative units was founded from the GIS database that was built for Wasit Governorate. The area of the administrative unit refers to the size of all activities and land uses in each unit; hence, we can use it as an indication of social activity size
3. The total population represents the density of the administrative unit that will directly affect the level of interaction among people. See Figs. 5 and 6; it is clear that the population consternate in the middle and north area of Wasit Governorate.
4. Access node indicates the size of the roads network in the administrative unit. The major road network passes through the central part of Wasit Governorate, as shown in Fig. 7 level in Wasit Governorate.
5. Order services to all central services in the social sector were mentioned (Abbass Jasim et al. 2017), like health, education, and universities (see Fig. 8). It is noticed that the middle sector has the most significant share. The north parts came in the second level. Besides, all quieter heads and the human resource of prominent institutes were settled in the center of the governorate.
6. Urban ratio displays the ratio of the urban area to the all-district area, showing how the district has centrality in people’s relationships; see Fig. 9. It is also noticed that the central district has a big ratio compared to other districts, except the northern one.

## Results and discussions

**Fig. 10:**
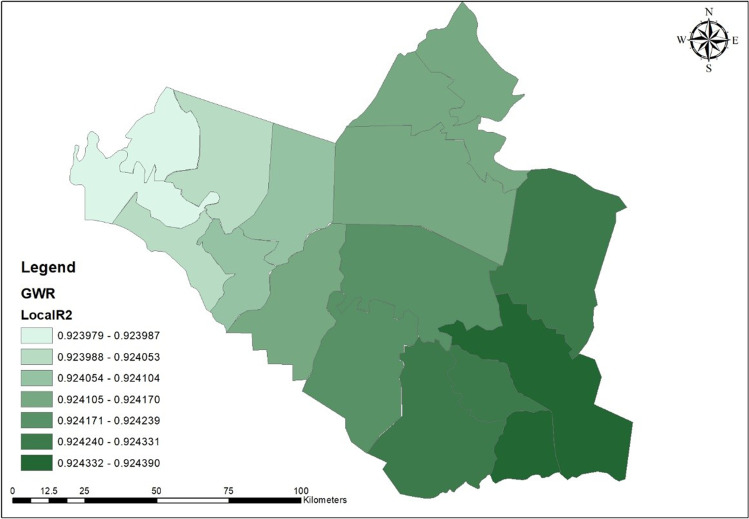
The local *R*^2^ value for Wasit Governorate

GWR explains the relationship between all independent variables and the dependent one. The value of true confidential relationship (*R*^2^) was 88, which means that the model cannot explain only 22 from all variables related to spatial dispersion COVID-19 pandemic. So, Fig. 10 shows the value of local (*r*^2^) for every district in the Wasit Governorate. The strength of the correlation of independent variables and their impact on infections was high and concentrated within seven administrative units out of 16 administrative units, with an estimated rate of 43% of the total number of administrative units, and constitute 42% of the total area of Wasit Governorate.

We can say that the level of confidence, explaining variables, and validity of the model is strong in the south of the governorate, and then decreased towards north and east, but it still strong until the middle of Wasit Governorate, where the highest COVID-19 infection was recorded.

**Fig. 11:**
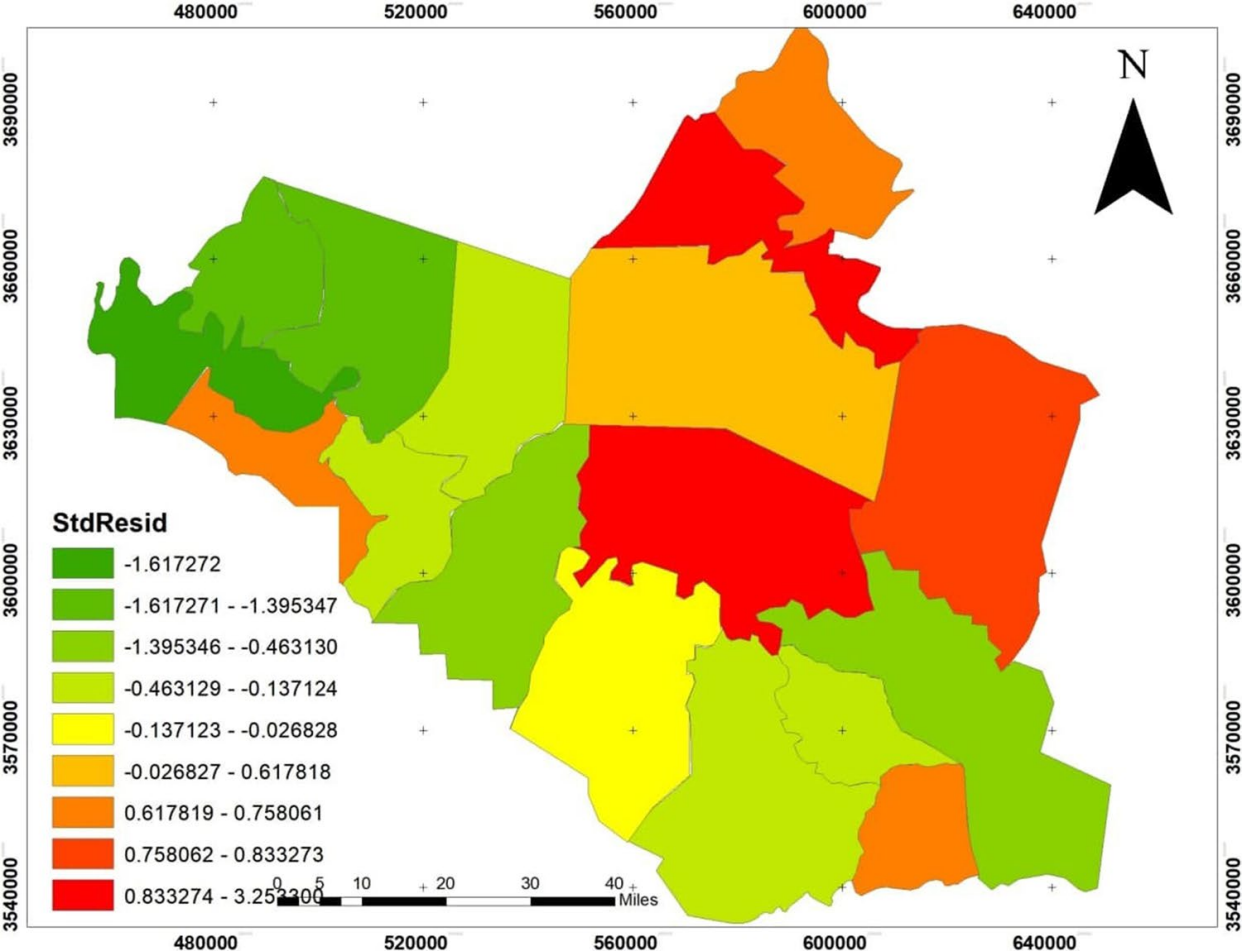
Distribution standard residuals to GWR model for the distribution of COVID-19 in Wasit Governorate

Figure 11 shows the reliability of data interpretation by analyzing the spatial distribution of standard deviation values. It showed that the administrative units in the south of the governorate were the most reliable, with the lowest standard deviation (less than − 1.617). In contrast, Badra and Kut administrative units have the highest standard deviation distribution values of 0.833–3.25. Kut administrative unit is the largest unit in terms of area, population density and transit traffic, a high order of social service system, and level of urbanization and being the administrative center of Wasit Governorate. Consequently, it included all the factors that increased the spread of the pandemic.

As for the values that were indicated less than the estimated value of the model, they were in Suwayrah district due to moderate movement and access, service system, urbanization, and population size (see Fig. 11).

The areas close to or less than the estimated value were in the south of Wasit Governorate and were characterized by the weakness of movement, the weakness of the hierarchy of social services provided; the size of the population is approximately average; and the rate of urbanization was close to the lowest value across the governorate.

**Fig. 12:**
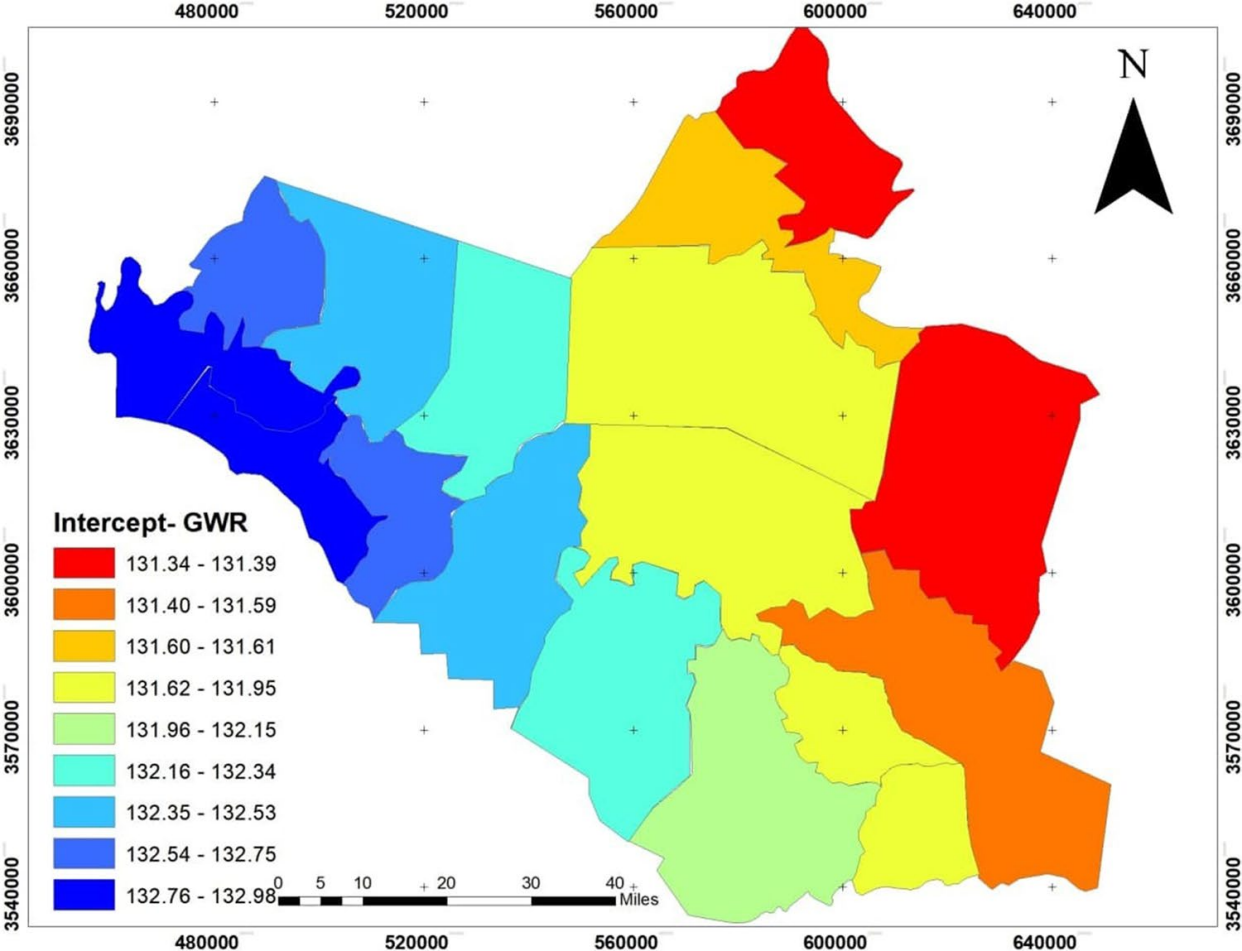
The value of GWR intercept in the districts of Wasit Governorate

Figure 12 shows that if the effects of the analyzed factors on the spread of the epidemic were neutralized, very different values would result as it becomes clear from the figure that the epidemiological infections are required to be confined to the northern region of Wasit Governorate, which is linked to the capital, Baghdad, within Units 8 in the northeast of Wasit Governorate and that the infections in the southern administrative units are generally within less than half of the infection affected. This shows that the GWR methodology was distinguished spatially between the factors affecting the spread of epidemic infections according to the factors chosen for testing with the number of infections for each administrative unit during 10 months of the epidemic.

In other words, if all factors have equal influence within all administrative units and neglect their effects, then infections will be more in the north than in the south of the governorate. Therefore, we can depend on the GWR result to clarify another spread pattern when other factors are in the model.

The model interprets other results as the direct positive relationship between the infection and each area, the level of urbanization, the total number of residents, and the movement level.

However, the relationship was inverse between the infections and the level of services provided by the administrative unit (Cvetković et al. 2020).

**Fig. 13:**
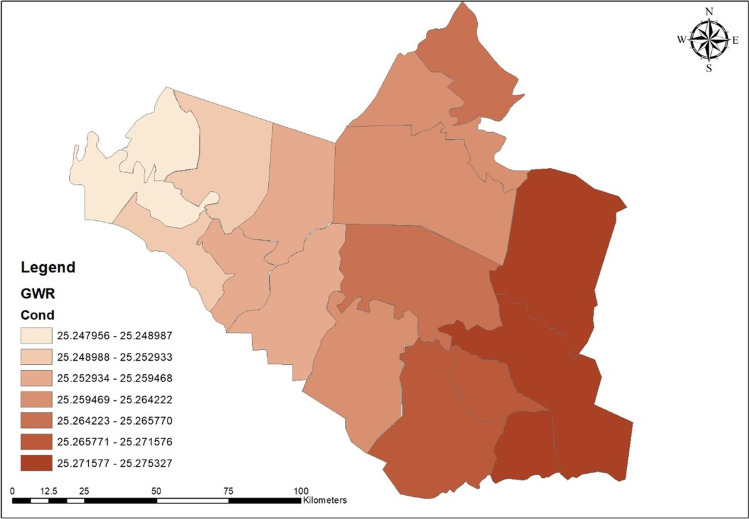
The value of GWR data reliability in Wasit Governorate

The number of conditions in the model was less than 30, indicating that reliable data was used. Figure 12 shows that the reliability is strong in the south and decreases to the governorate’s north (Fig. 13).

The COVID-19 pandemic has evolved quickly into one of the most threatening and devastating public health crises in recent history (Mansour et al. 2021). While most COVID-19 studies have been undertaken from a medical perspective or focused on epidemiological evolution, there is a growing literature applying spatial analysis and disease mapping, particularly in the developed world. The key findings from this research were that a set of physical, social, and economic variables were found to impact the COVID-19 spreading rate and that these factors vary geographically. More specifically, the model results indicated a considerable spatial heterogeneity in COVID-19 spreading rates along the urbanization gradient. The higher infection rates were at the north and associated with higher population densities, consistent with previous studies that reported the positive influence of overcrowding and population densities on transmission rates. These studies have found a positive association of the population density in built-up areas for many researches (Lak et al. 2021; Li et al. 2021). At the same time, the ratio of the urban population has a significant positive relation towards recovery rate (Hassan et al. 2020; Ramírez-Aldana et al. 2020). It is clear. The low level of infection in the north and east areas of Wasit Governorate is related to the low level of air pollution in these areas, especially since these areas are characterized by the nature of dense agriculture, palm trees, and orchards.

It is well known that green spaces and dense agricultural areas greatly help purify the air, reduce pollution, and reduce the severity number of infections. And this is consistent with what was confirmed by the Bilal study regarding air pollution in several parts of Germany, as attention to clean air and its freedom from pollutants had qualitative and quantitative results on the decline of epidemic infection in Germany.

Hence, addressing the social factors that create poor health is essential to reducing inequities in the health impacts of disasters.

## Conclusions and policy implications

This study aims to search the relationship between society’s physical, social, and economic characteristics and coronavirus (COVID-19) pandemic spread using the GWR model. The results showed that the administrative units in the south of the governorate were the most reliable, with the lowest standard deviation (less than − 1.617). In comparison, Badra and Kut administrative units have the highest standard deviation distribution values ranging between 0.833 and 3.25. Kut administrative unit is the largest unit in terms of area, population density and transit traffic, a high order of social service system, and level of urbanization and being the administrative center of Wasit Governorate. Consequently, it included all the factors that increased the spread of the pandemic.

High population density is closely related to the spread of the epidemic and the number of infections as the highest population density areas has the highest number of infections. Moreover, the GWR model has proved a direct and positive relationship between infections and the administrative unit area. Likewise, a direct positive relationship between the infections and the level of urbanization in each administrative unit has been revealed.

It can be concluded that the low level of infection in the north and east areas of Wasit Governorate is related to the low level of air pollution in these areas, especially since these areas are characterized by the nature of dense agriculture, palm trees, and orchards, and as it is known that green areas and dense agricultural areas greatly help to purify the air and reduce pollution, thus reducing the infections and its severity.

Because the comprehensive ban has a negative impact on the economy and social relations, it is sometimes helpful to disable the factors affecting the increase in the strength of the pandemic without adopting a comprehensive ban policy in administrative units. Hence, through the model results, it will be possible to deal with each administrative unit in proportion to its circumstances in light of the factors affecting it.

Despite the development of many vaccines, the continuous spread of the pandemic and the reluctance of many people in developing countries to take it require taking measures to limit its spread. Therefore, there is a need for further research on spatial modelling of disease transmission and dynamics at the community level to identify potential drivers that may influence infection rates and investigate the complex links between these factors.

In light of this, it is also possible to adopt a set of preventive methods based on the elements of the spatial interaction of urban areas, such as emphasis on imposing health care on low-income areas due to the emergence of more infection due to their violation of the comprehensive ban due to their economic situation. Moreover, a culture of remote electronic communication, whether in work or education, can reduce the possibility of epidemic infections if the technical infrastructure is provided. It is also possible to reduce transit traffic from outside the areas and limit mixing with strangers to limit infections outside the specified area. In addition to encouraging burrowing and cultivation in any available place, it has been proven that it has a role in reducing the various levels of pollution and increasing the immunity of human health in anticipation of any future epidemics.

## Data Availability

Not applicable.
